# CT-derived skeletal muscle change before immunotherapy predicts survival of advanced gastric cancer: associations with inflammatory markers and liver lipid metabolism

**DOI:** 10.1007/s10147-024-02551-x

**Published:** 2024-05-22

**Authors:** Koichi Hayano, Gaku Ohira, Yasunori Matsumoto, Yoshihiro Kurata, Ryota Otsuka, Atsushi Hirata, Takeshi Toyozumi, Kentaro Murakami, Masaya Uesato, Hisahiro Matsubara

**Affiliations:** Department of Frontier Surgery, Chiba University Graduate School of Medicine, 1-8-1 Inohana, Chuo-Ku, Chiba, 260-8677 Japan

**Keywords:** Skeletal muscle, Gastric cancer, Immune checkpoint inhibitor, Systemic inflammation, Liver lipid metabolism

## Abstract

**Background:**

Skeletal muscle (SM) is a key factor in cancer treatment. However, it is unclear whether pretreatment SM change affects the outcome of immune checkpoint inhibitors (ICIs) therapy in gastric cancer (GC).

**Methods:**

Advanced GCs treated with ICIs were retrospectively investigated. SM evaluated by psoas muscle area at the third lumbar vertebra was measured on CT acquired within 1 month from the start of ICIs therapy (CT-1), and on CT acquired 2.8 ± 0.84 months before CT-1. Monthly change rate of SM (MCR-SM) was defined as the change rate of SMs between those two CTs divided by the period between those CTs (month). Monthly change rate of body weight (MCR-BW) during the same period was also calculated. They were compared with disease-specific survival (DSS) and progression-free survival (PFS). MCR-SM was compared with pretreatment markers including neutrophil-to-lymphocyte ratio (NLR), platelet-to-lymphocyte ratio (PLR), monocyte-to-lymphocyte ratio (MLR), C-reactive protein (CRP), and liver-to-spleen CT attenuation ratio (LSR) as a marker of liver lipid metabolism.

**Results:**

This study enrolled eighty-three GC patients. MCR-SM significantly correlated with DSS and PFS (*P* < 0.0001, 0.001, respectively), whereas MCR-BW did not. Kaplan–Meier analyses demonstrated that higher MCR-SM (MCR-SM ≥ −0.7185%) significantly associated with better DSS and PFS (*P* = 0.0002, 0.03, respectively). Patients with positive MCR-SM showed significantly lower NLR, MLR, and CRP than those with negative (*P* = 0.01, 0.006, 0.003, respectively). MCR-SM showed a significant positive correlation with LSR (*P* = 0.007, *R* = 0.30).

**Conclusions:**

Pretreatment SM loss, associated with high systemic inflammation and hepatic fat accumulation, related to poor outcome of ICIs therapy in GC.

## Introduction

Gastric cancer (GC) is the fifth common cancer and the third leading cause of cancer-related death worldwide [1]. Since the ATTRACTION-2 study [2] and the KEYNOTE-059 study [3], immune checkpoint inhibitors (ICIs) have been recommended for previously treated unresectable advanced or recurrent gastric cancer. However, those studies reported that the 1-year overall survival (OS) rate of ICIs therapy, such as nivolumab and pembrolizumab, was 23.2–26.2% [2, 3]. It means that about 75% of patients die within 1 year, although ICIs therapy is obviously better than placebo. Therefore, investigation of biomarkers or clues to improve treatment response of ICIs are highly desirable.

On the other hand, it has been reported that cachexia is associated with poor prognosis in any types of cancer, and a typical symptom of cachexia is loss of skeletal muscle (SM) [4]. In nivolumab treatment of advanced GC patients, a recent study reported that the measurement of SM mass index at the time of starting ICIs therapy might be useful for predicting treatment outcome [5]. However, it is still unclear whether the degree of SM change before ICIs therapy can predict treatment outcome of ICIs therapy for advanced GC. If SM change before ICIs therapy really has an impact on the outcome of ICIs therapy, we may be able to find a new strategy to enhance ICIs therapy from the viewpoint of SM intervention.

Therefore, this study investigated whether SM change before ICIs therapy was associated with prognosis of ICIs therapy in advanced GC, compared with various cachexia related markers, such as systemic inflammatory markers or liver lipid metabolism [6–8].

## Materials and methods

### Patient population

This retrospective study was performed under the approval of the institutional review board at our institute (IRB No. 3032). Written informed consent for participation was waived because of the retrospective nature of this study. We retrospectively identified unresectable advanced or recurrent gastric cancer patients treated with ICIs (nivolumab or pembrolizumab) in our hospital from October 2017 to June 2023. All patients received at least first-line chemotherapy prior to ICIs therapy.

### Treatment and follow-up

The treatment schedule and the dose modification schema of ICIs therapy using nivolumab and pembrolizumab have been detailed previously [2, 9]. Nivolumab was administered intravenously at a dose of 240 mg every 2 weeks, and pembrolizumab was administered intravenously at a dose of 200 mg every 3 weeks. These treatments were continued until disease progression, unacceptable toxicity, or patient refusal. Tumor responses were assessed by CT every 2–4 cycles of ICIs therapy. After the failure of ICIs therapy, any additional treatment occurred at the discretion of the treating physician, but basically conformed to the Japanese gastric cancer treatment guidelines [10].

### Assessment of skeletal muscle change and body weight change

**Fig. 1:**
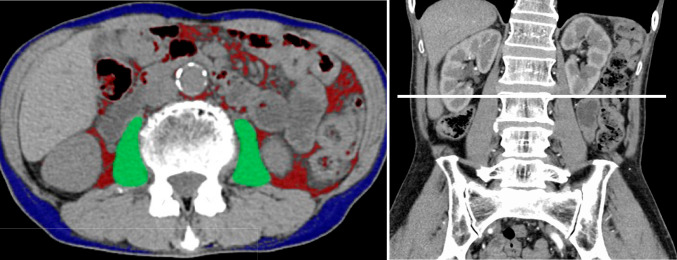
Skeletal muscle was evaluated by the areas of psoas muscle at the third lumbar vertebra level on CT image, and the area of PM was automatically drawn using Synapse Vincent (green area)

Patients were examined on 320/80-section multi-detector row CT (MDCT) scanners (Aquilion ONE VISION/Aquilion PRIME; Canon Medical Systems, Otawara, Japan). The following CT parameters were used for acquisition of volume data: 100 kVp; 150–200 mA; 0.5-s rotation time; field of view, 360; matrix, 512; pixel size, 0.7 mm × 0.7 mm; 5 mm reconstructed slice thickness. SM was evaluated by the areas of psoas muscle (PM) at the third lumbar vertebra on CT image, and this PM area within a range of −29 to 150 Hounsfield units was automatically identified using Synapse Vincent (Fujifilm Inc., Tokyo, Japan) (Fig. 1). PM was measured on CT acquired within 1 month from the start of ICIs therapy (CT-1), and on CT acquired at 2.8 ± 0.84 months before CT-1. Monthly change rate of SM (MCR-SM) was defined as the change rate (%) of PMs between those two CTs at different timepoints divided by the period between those CTs (month). Monthly change rate of body weight (MCR-BW) was also calculated as the change rate of body weights between those two CTs divided by the period between those CTs (month).

### Systemic inflammatory markers’ measurements before ICIs therapy

Systemic inflammatory markers were calculated as following; neutrophil-to-lymphocyte ratio (NLR) was calculated by dividing absolute neutrophil count by lymphocyte count, platelet-to-lymphocyte ratio (PLR) was by dividing thrombocyte count by lymphocyte count, and monocyte-to-lymphocyte ratio (MLR) was by dividing monocyte count by lymphocyte count measured in peripheral blood before ICIs therapy. Serum C-reactive protein (CRP) was also measured in peripheral blood before ICIs therapy.

### Assessment of liver lipid metabolism

Liver lipid metabolism was assessed by non-contrast enhanced CT acquired within 1 month from the start of ICIs therapy (CT-1). Mean CT attenuation values were obtained for both the liver and the spleen using a single oval region-of-interest (ROI) of about 200 mm^2^, taking care to avoid vessels, calcifications, and masses, if present. Thereafter, liver-to-spleen ratio (LSR; liver attenuation/spleen attenuation) was calculated as the quantitative marker for liver lipid metabolism [11, 12].

## Statistical analysis

Statistical analyses were performed using the JMP Pro 16.0 (SAS Institute, Inc., Cary, NC, USA), and for all comparisons, *P* < 0.05 was considered to indicate a statistically significant difference. The associations of the continuous variables with disease-specific survival (DSS) and progression-free survival (PFS) after the ICIs therapy were evaluated using the Cox proportional hazards regression model. Kaplan–Meier analysis was also performed for DSS and PFS analyses, and the log-rank test was employed. The Mann–Whitney U test was applied for comparison of inflammatory markers between positive MCR-SM and negative MCR-SM groups. Correlations between MCR-SM and LSR was analyzed using Spearman’s rank correlation coefficients.

## Results

### Patient characteristics

Eighty-three patients who were treated with ICIs in our hospital were eligible for this study. This subject included 56 men and 27 women, with a median age of 70.0 years (range 23–87 years). Seventy-nine cases were treated with Nivolumab, while 4 cases were treated with Pembrolizumab. Patients’ characteristics and their associations with MCR-SM are summarized in Table 1. Higher MCR-SM was significantly associated with older age (≥71), recurrence GC rather than unresectable GC, higher BMI at ICIs therapy (≥19.72%), higher MCR-BW (≥ −0.231%), IrAEs occurrence, higher serum albumin (≥3.4), and higher prognostic nutrition index (≥ 39.815) (*P* = 0.006, 0.03, 0.008, 0.01, 0.01, 0.004, 0.008, respectively).

**Table 1:** Patient characteristics

Patient demographics	Variables (*n*)	MCR-SM (%)	*P*
Sex	Male	−1.681 ± 6.973	0.8
	Female	−1.505 ± 6.014	
Age	<71 (42)	−3.469 ± 5.781	0.006*
	≥71 (41)	0.390 ± 6.274	
ECOG PS	0 (31)	−0.181 ± 4.305	0.059
	1 (52)	−2.386 ± 7.145	
Type of cancer	Recurrence (45)	−0.178 ± 6.055	0.03*
	Unresectable (38)	−3.202 ± 6.265	
Number of prior regimens	1, 2 (78)	−1.652 ± 6.187	0.9
	3, 4 (5)	−0.171 ± 8.603	
ICIs	Nivolumab (79)	−1.777 ± 6.171	0.2
	Pembrolizumab (4)	2.684 ± 8.312	
BMI at ICIs therapy	<19.72 (41)	−3.339 ± 6.839	0.008*
	≥19.72 (42)	−0.170 ± 5.243	
MCR-BW (%)	<−0.231 (42)	−3.131 ± 6.380	0.01*
	≥−0.231 (41)	0.043 ± 5.865	
Histology	Diffuse (44)	−2.137 ± 6.406	0.2
	Intestinal (39)	−0.914 ± 6.195	
IrAEs	Yes (22)	0.988 ± 4.190	0.01*
	No (61)	−2.483 ± 6.696	
Treatment response	Complete or partial response (13)	0.683 ± 3.678	0.051
	Stable or progressive disease (70)	−1.980 ± 6.609	
Serum albumin (g/dl)	<3.4 (41)	−3.380 ± 5.444	0.004*
	≥3.4 (42)	0.211 ± 6.627	
Prognostic nutrition index	<39.815 (41)	−3.120 ± 6.103	0.008*
	≥39.815 (42)	−0.042 ± 6.183	

*MCR-SM* monthly change rate of skeletal muscle, *ECOG PS* Eastern Cooperative Oncology Group Performance Status, *ICIs* immune checkpoint inhibitors, *BMI* body mass index, *MCR-BW* monthly change rate of body weight, *IrAE* immune-related adverse event; Cut-off values were based on median values; *, significant difference at *P* < 0.05

### Correlations of skeletal muscle change with survival

**Fig. 2:**
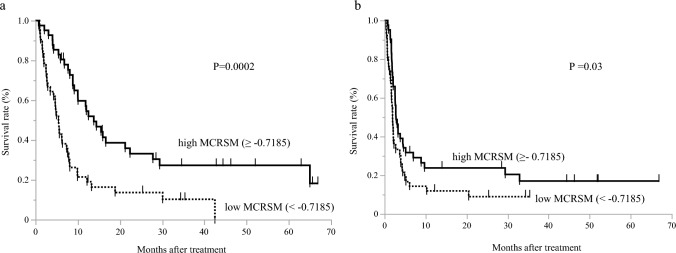
Kaplan–Meier analyses demonstrated that patients with higher MCR-SM (≥ -0.7185%, median) showed significantly better disease-specific survival (**a**) and progression-free survival (**b**)

In univariate Cox regression analysis (Table 2), MCR-SM showed significant correlations with DSS and PFS (*P* < 0.0001, 0.001, respectively), but MCR-BW did not (*P* = 0.08, 0.2, respectively). Kaplan–Meier analyses demonstrated that patients with higher MCR-SM (MCR-SM ≥ −0.7185%, median value) had significantly better DSS and PFS (P = 0.0002, 0.03, respectively; Fig. 2).

**Table 2:** Univariate Cox regression analyses for disease-specific survival and progression-free survival

	Disease-specific survival			Progression-free survival		
Variables	HR	95%CI	*P*	HR	95%CI	*P*
Sex	1.070	0.631–1.813	0.8	0.929	0.563–1.531	0.7
Age	0.982	0.966–1.001	0.06	0.976	0.960–0.994	0.01*
MCR-SM	0.887	0.846–0.932	<0.0001*	0.930	0.891–0.972	0.001*
MCR-BW	0.906	0.819–1.013	0.08	0.936	0.843–1.048	0.2

*MCR-SM* Monthly change rate of skeletal muscle, *MCR-BW* Monthly change rate of body weight; *Significant difference at *P* < 0.05

### Associations of skeletal muscle change with systemic inflammatory markers and liver lipid metabolism

**Fig. 3:**
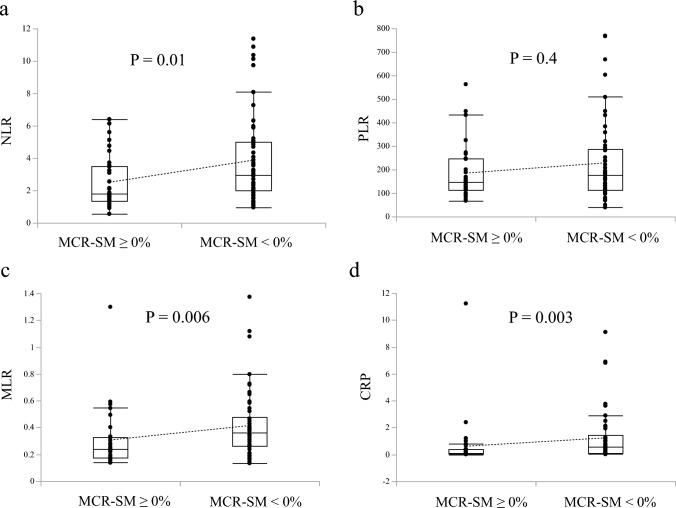
Patients with positive MCR-SM showed significantly lower NLR (**a**), MLR (**c**), and CRP (**d**) values than those with negative, whereas PLR (**b**) did not show a significant association with MCR-SM

**Fig. 4:**
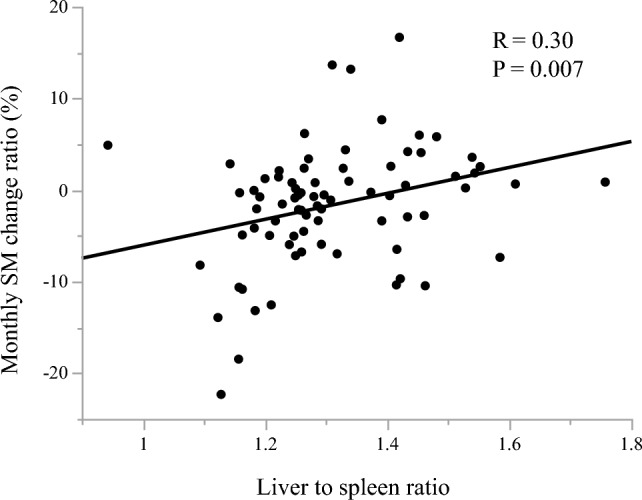
MCR-SM showed a significant positive correlation with LSR

This study cohort included 33 patients with positive MCR-SM (MCR-SM ≥ 0%) and 50 with negative MCR-SM (MCR-SM < 0%). Patients with positive MCR-SM showed significantly lower NLR, MLR, and CRP values than those with negative MCR-SM (*P* = 0.01, 0.006, 0.003, respectively), whereas PLR did not show a significant association with MCR-SM (*P* = 0.4, Fig. 3). MCR-SM showed a significant positive correlation with LSR (*P* = 0.007, *R* = 0.30; Fig. 4.).

## Discussion

A meta-analysis including 2501 patients with solid cancers reported that sarcopenia could predict the response to ICIs and survival after ICIs therapy [13]. Another meta-analysis demonstrated that not only the baseline sarcopenia but the development or worsening of sarcopenia during immunotherapy associated with poor overall survival in advanced non-small cell lung cancer [14]. In ICIs therapy of GC, three previous studies reported that low baseline SM or sarcopenia was associated with poor survival after ICIs therapy, and they are summarized in Table 3 [5, 15, 16]. Therefore, SM loss, which is a typical symptom of cachexia, obviously plays an important role in ICIs therapy. However, these previous studies focused on SM index or PM index at the time of starting ICIs therapy, or the change of SM or PM index “after” ICIs therapy, and it was still unclear whether the degree of SM change prior to ICIs therapy affected the outcome of ICIs therapy. In this context, our study demonstrated that SM change rate before ICIs therapy obviously affected the outcome of ICIs therapy. Considering these results including ours, prevention of SM loss before and during ICIs therapy is quite important to make ICIs therapy more effective. The prognostic importance of SM loss was also reported in the first-line chemotherapy of GC [15]; therefore, we strongly recommend keeping SM in both immunotherapy and cytotoxic chemotherapy of GC with an intervention on SM or nutrition approach. Furthermore, a meta-analysis demonstrated that preoperative SM loss increased the risk of postoperative complications and reduced the overall survival rate of patients undergoing gastrectomy for GC [17]. Of course, some patients with severe cachexia such as refractory cachexia, which was categorized by Fearon et al. in 2011 [18], might be resistance to exercise or nutrition intervention, because it was reported that those at refractory cachexia were resistant to any type of intervention due to strong catabolic effects [19]. However, it is still difficult to precisely distinguish patients with refractory cachexia from those with other cachexia categories such as precachexia and cachexia who may have a chance to improve their prognosis with exercise or nutrition intervention to keep SM [20–22]; therefore, it would be better for all GC patients to start exercise or nutrition intervention to prevent SM loss as soon as they are diagnosed as GC at any stage, if they want to improve their treatment outcome.

**Table 3:** Summary of previous studies on skeletal muscle and survival in gastric cancer patients treated with ICIs

	Year	Number of patients	Association of skeletal muscle or sarcopenia with survival
Fujii et al. [16]	2020	44	Baseline sarcopenia was associated with poor OS
Kim et al. [15]	2021	149	Baseline sarcopenia was associated with poor PFS
Kano et al. [5]	2021	31	Low baseline PMI was associated with poor PFS

*ICIs* immune checkpoint inhibitors, *OS* overall survival, *PFS* progression-free survival, *PMI* psoas muscle index

This study also compared SM change with systemic inflammatory markers and liver lipid metabolism, and demonstrated that short-term SM loss prior to ICIs therapy was associated with high systemic inflammation and hepatic fat accumulation (lower LSR) before ICIs therapy. Generally, inflammatory markers, such as NLR, PLR, LMR, and CRP, were reported their associations with cancer cachexia [23–25], and some previous reports demonstrated that the elevations of these inflammatory markers were associated with poor outcome of cancer patients treated with ICIs therapy [26–29]. On the other hand, our previous study demonstrated that ICIs were ineffective in GC patients with severe hepatic steatosis [6]. Similarly, Pfister et al. [30] reported that ICIs were ineffective in non-alcoholic steatohepatitis (NASH)-related hepatocellular carcinoma. Pfister et al. [30] suggested that NASH-related aberrant T cell activation caused tissue damage leading to impaired immune surveillance. Our previous study suggested that hepatic steatosis was associated with cancer cachexia leading to poor outcome in ICIs therapy [6]. Considering the results of these previous reports, it was reasonable that SM loss before ICIs therapy, which had significant associations with high systemic inflammation and hepatic fat accumulation, showed a significant correlation with worse survival in ICIs therapy of GC. However, further investigation will be needed to confirm this relationship.

Our study has several limitations. First, our findings are based on single-center data, and the sample size was small; therefore, this study should be validated in a larger patient population. Second, the period to measure SM change was varied in each individual case, due to the retrospective nature of this study, which can be a bias of this study. Third, ROIs for liver and spleen were manually drawn, and this procedure for measurement of LSR might be relatively subjective. A computerized new segmentation method with high reproducibility and reliability should be investigated in further studies.

## Conclusion

Though this was a relatively small study, it was demonstrated that short-term SM loss prior to ICIs therapy was associated with poor survival in GC patients. Exercise or nutrition intervention to prevent SM loss before ICIs therapy might improve the outcome of ICIs therapy. We believe that our results will provide an important insight into selecting the optimal therapeutic strategy for patients with advanced GCs.

## Declarations

### Data availability statement

The data that support the findings of this study are available from the corresponding author upon reasonable request.
